# A touching advantage: cross-modal stop-signals improve reactive response inhibition

**DOI:** 10.1007/s00221-023-06767-7

**Published:** 2024-01-16

**Authors:** Maximilian A. Friehs, Philipp Schmalbrock, Simon Merz, Martin Dechant, Gesa Hartwigsen, Christian Frings

**Affiliations:** 1 Psychology of Conflict, Risk and Safety, Department of Technology, Human and Institutional Behaviour, Faculty of Behavioural, Management and Social Sciences, University of Twente, Enschede, The Netherlands; 2 Department of General Psychology and Methodology, Trier University, Trier, Germany; 3 UCLIC, University College London, London, UK; 4 School of Psychology, University College Dublin, Dublin, Ireland; 5 Lise-Meitner Research Group Cognition and Plasticity, Max Planck Institute for Human Cognitive and Brain Sciences, Leipzig, Germany; 6 Wilhelm Wundt Institute for Psychology, Leipzig University, Leipzig, Germany; 7 ZEISS Vision Science Lab, Carl Zeiss Vision International GmbH, Turnstrasse 27, 73430 Aalen, Germany

**Keywords:** Inhibition, Stop-signal, Distractors, Cross-modal

## Abstract

The ability to inhibit an already initiated response is crucial for navigating the environment. However, it is unclear which characteristics make stop-signals more likely to be processed efficiently. In three consecutive studies, we demonstrate that stop-signal modality and location are key factors that influence reactive response inhibition. Study 1 shows that tactile stop-signals lead to better performance compared to visual stop-signals in an otherwise visual choice-reaction task. Results of Study 2 reveal that the location of the stop-signal matters. Specifically, if a visual stop-signal is presented at a different location compared to the visual go-signal, then stopping performance is enhanced. Extending these results, study 3 suggests that tactile stop-signals and location-distinct visual stop-signals retain their performance enhancing effect when visual distractors are presented at the location of the go-signal. In sum, these results confirm that stop-signal modality and location influence reactive response inhibition, even in the face of concurrent distractors. Future research may extend and generalize these findings to other cross-modal setups.

## Introduction

Stopping an already initiated response is vital for adaptive everyday behavior. For example, a pedestrian might have to stop before a crosswalk when the traffic light suddenly changes to red or a cook might have to stop reaching towards the stovetop, as soon as they realize it is still hot. These examples represent only two situations in which people have to withhold a response to achieve a goal once a change of information appears. In the laboratory, the ability to inhibit already initiated responses can be measured using tasks such as the Stop-Signal Task (SST) (Verbruggen et al. 2019).^1^ In the typical SST, a participant is required to perform a choice-reaction time task, for example, pressing a button on the left or right sides depending on the orientation of an arrow, and on a random subset of trials, a cue instructs the participants to withhold their response. This stop-signal is usually visual or auditory, but it is unclear how performance changes in response to tactile stop-signals. For example, a study by Ikarashi et al. (2022) compared visual, auditory and tactile signals in the SST. The data revealed no difference in reactive stopping depending on the signal type. However, they did not employ a cross-modal setup. In other paradigms such as stop-change or multitasking, multimodal information may heavily influence reaction speed. For example, simultaneous multimodal input can increase attention towards the stimulus, with tactile-visual stimuli resulting in a larger impact (Gohil et al. 2016; Stock et al. 2017). Thus, it seems clear that multimodal signals may not necessarily be advantageous (Strelnikov et al. 2021). However, albeit both visual and auditory stop-signals are often used, stopping performance in unimodal versus cross-modal (e.g., stop- and go-signals are not presented in the same modality) has rarely been evaluated systematically. Some research suggests that auditory stop-signals in an otherwise visual SST enhance speed and efficiency of stopping (Van Der Schoot et al. 2005). This effect seemed to be further increased for louder noises compared to quieter ones (see also Ramautar et al. 2006). In this regard, it has been put forward that the inhibition process in the SST can be divided into two processing stages: (1) a detection and encoding stage followed by (2) an inhibition and interruption stage (Boucher et al. 2007; Logan 2015; Verbruggen and Logan 2015). Thus, the advantage of auditory signals could either be linked to one stage or the other. Recent evidence suggests that the modality affects both stages of stopping (Carrillo-de-la-Peña et al. 2019). Against the background of the aforementioned research, it may be hypothesized that the effects observed for auditory stop-signals might be transferred to the tactile modality, because a cross-modal stop-signal may generally enhance performance in an otherwise unimodal setup (i.e., a tactile stop-signal in an otherwise visual task). Yet, this question bears importance not just on the theoretical, but also on a practical level. To increase our understanding of the mechanisms underlying response inhibition, it is important to define the relevant boundary conditions under which inhibition performance changes. Modality effects have also been evaluated in other tasks that require inhibition such as, for example, the negative priming paradigm (for an extensive discussion, see Frings et al. 2015). In short, the negative priming paradigm requires the participant to respond to a specific stimulus feature while ignoring other features of the stimulus. If an ignored feature is repeated as the target in a subsequent stimulus, performance is worse, because the feature as well as its associated response had just been inhibited. Although such effects are observable across all modalities, the tactile negative priming effect is larger compared to the effect in vision (Frings et al., 2011). Although the negative priming task and the SST are different, both require cognitive and behavioral inhibition. Yet, also on a practical level, the question regarding tactile response inhibitory signals is of utmost interest. That is, while we strongly rely on visual and auditory input to perceive our environment to act sensibly and safely (think about driving a car—one relies heavily on visual and auditory input to scan the surroundings and drive safely), the tactile modality is rather neglected in these contexts. If inhibitory processes can be initiated by tactile stimulation, this would call for the development of touch-based inhibitory systems. In fact, as it comes to presenting warning signals to initiate a response, such as a braking response, previous research has shown the advantages of the tactile modality for faster responding (for an extensive discussion, see Meng and Spence 2015). Yet, for these studies, the central objective was to initiate a response—if such and related results extend to the inhibition of a planned response is the objective of the present study.

## The present study

In this manuscript, we report upon three separate studies exploring the effects of stop-signal modality and the robustness of performance enhancement via cross-modal stop-signals even in distractor rich environments. To elaborate, study 1 contrasted tactile and visual stop-signals in an otherwise visual choice-reaction time task. Tactile stop-signals were presented via vibrations on the left hand, while visual stop-signals were presented on the screen in front of the participants. We hypothesized that general reaction speed and error rates are not affected by stop-signal modality. Yet, if cross-modal stop-signals are generally processed more effectively, the inhibition process (as measured by stop-signal reaction time; SSRT) should be enhanced in the tactile task condition as compared to the visual task condition. However, this experimental setup entails a potentially problematic confounding variable: the stop-signal position. Specifically, the visual stop-signal is presented at the same location as the go-signal, while the tactile stop-signal is presented on the participants’ hand and not linked to the go-signal. Thus, study 2 utilized a slightly different experimental setup in relation to how the stop-signals are presented. In short, in study 2 (and 3 as well), the stop-signals were presented using a multisensory cube through which visual as well as tactile stimuli can be presented. These cubes are comfortable to hold for a participant and equipped with LEDs and tactors. Importantly, this ensures that both visual and tactile stop-signals are presented at the same spatial location. Further, this spatially discriminates between go- and stop-signals altogether. We hypothesized that for tactile stop-signals, study 2 would replicate results from study 1, while disentangling the location of visual stop- and go-signals should positively influence performance. Despite these findings, questions lingered regarding the robustness of these effects against distractions in visually noisy environments. Consequently, Study 3 delved into the impact of visual distractors on SST performance, employing either tactile or visual stop-signals. This study is both important from a theoretical as well as practical perspective. From a theoretical viewpoint, as perceptual processes are key to stopping, a perceptual load manipulation via distractors may influence performance (Verbruggen et al. 2014). After all, “the first step in successfully cancelling a response is nearly always detecting the stop signal” (Verbruggen et al. 2014, p. 1296). There has been evidence that distractions result in stopping and general performance deficits (Chambers et al., 2007; Verbruggen et al., 2004, 2014). However, detection of the stop-signal in perceptually demanding environments may potentially be supported by either disentangling stop- and go-signal location or using a tactile stop-signal. Thus, while we expected the replication of previous findings favoring tactile stop-signals, we were cognizant of potential negative performance influences of visual distractors. Nevertheless, if the location of the stop-signal matters, then performance may not drop drastically in the visual stop-signal condition as well as the in the tactile stop-signal signal condition. Put differently, if disentangling stop- and go-signals generally improves performance, then distractors at the location of the go-signal should not influence processing of the stop-signal and study 2 should be replicated. Consequently, from a practical standpoint, if previous findings generalize to visual distractors, this has implications for the application of tactile stop-signals in human-technology interfaces (e.g., as tactile warning signals for machine operators). Thus, in predominantly visually demanding tasks, a visual or tactile warning signal may be most effective if presented spatially disentangled from the main visual focus point. After all, in everyday life, stop-signals oftentimes occur in distracting environments, and thus, the ability to detect a relevant stop-signal among perceptual distractors is key to successful stopping.

## Study 1: contrasting visual and tactile response inhibition

### Method

#### Sample

Based on previous research (Verbruggen et al. 2014; Wessel and Aron 2014), we expect at least a medium-sized effect. For *f* = 0.5, *α* = 0.05, and power 1 − *β* = 0.95, a sample of at least 16 people is needed assuming a correlation between the four conditions of at least *r* = 0.5. To test also for smaller effects, as well as to potentially deal with dropout or low-quality data due to participants not understanding the task, we collected data from a larger sample of 24 participants (20 female, 4 male, aged 19–27) with a mean age of 21.92 (SD = 2.64). We assessed gender via self-report. Options given were the same across all experiments: female, male, non-binary, prefer to self-report, and prefer to not disclose. All participants were right-handed.

#### Design

The study had a repeated-measures design to evaluate the effect of stop-signal modality on performance within participants. Thus, we used a 2 (stop-signal modality: *visual* vs. *tactile*) × 2 (order: *visual first* vs. *tactile first*) mixed design, with the order being counterbalanced between participants.^2^ The main dependent variable was the Stop-Signal Reaction Time (SSRT, i.e., the estimate of time needed to respond to the Stop-signal and to cancel the movement), which is a measure of the reactive inhibition process. Every participant completed two SST blocks in one sitting: one with a visual and one with a tactile stop-signal. The order was counterbalanced across participants.

#### Materials and procedure

**Fig. 1:**
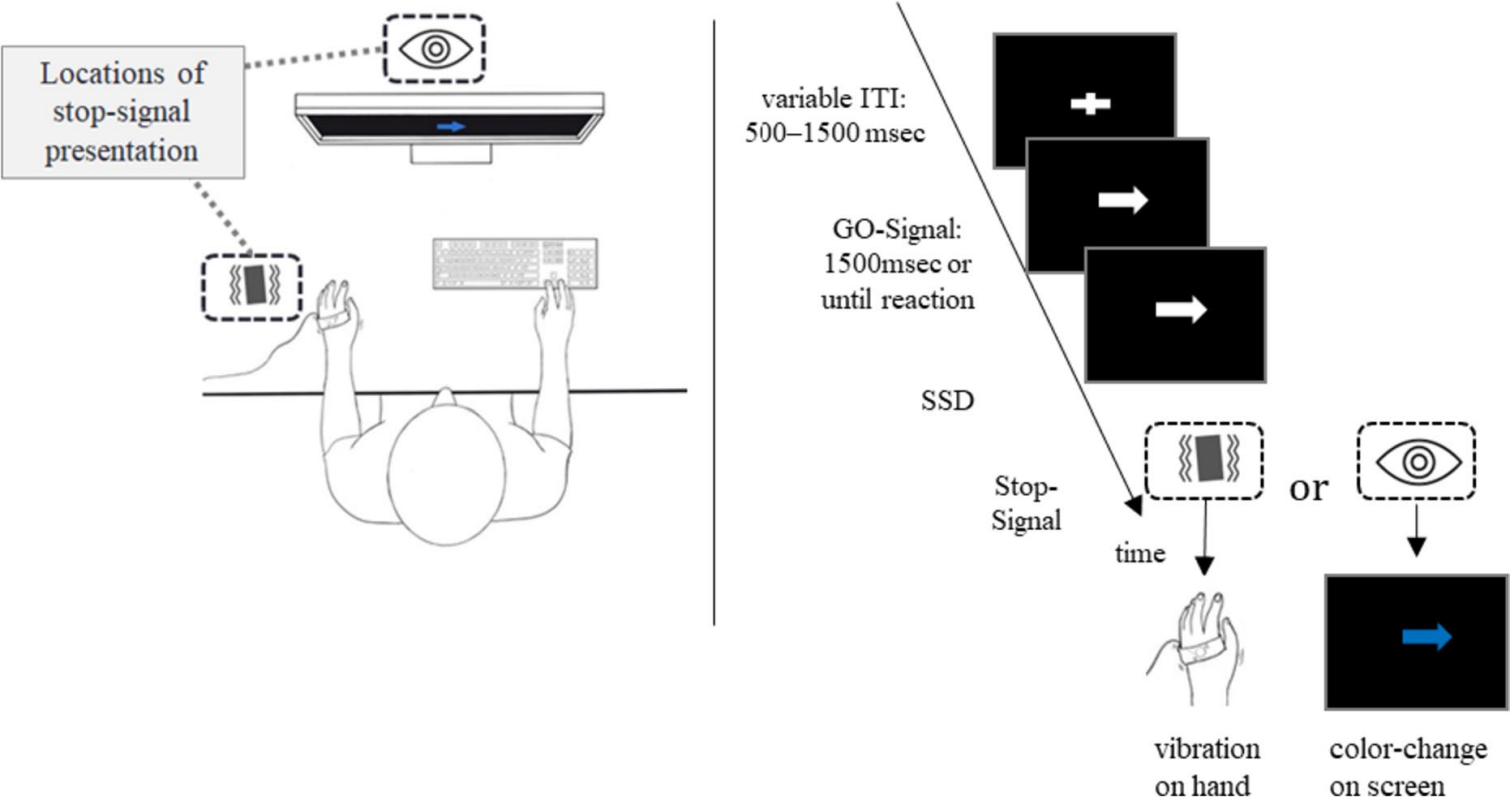
Left: experimental setup for study 1. Participants were seated in a dark, sound-attenuated room in front of a screen at a comfortable viewing distance of ~ 60 cm. The stop-signal was either tactile (vibrations on the back of the participant’s hand) or visual (blue coloration of the arrow). Right: exemplary trial sequence

Upon entering the laboratory, participants were randomly assigned to one of two order conditions: tactile or visual stop-signal task first. Participants were tested individually in a dark, sound-attenuated room with a viewing distance of approx. 60 cm to the screen. The participant’s left forearm was stationary with a tactor strapped to the back of their hand (Model C-2, Engineering Acoustic, Inc.; controlled via the serial interface). The participants wore earplugs (noise reduction: 29 dB) on top of which brown noise was presented over headphones (over-ear headphones: ~ 85 dB). Both earplugs and the brown noise were present for both stop-signal conditions. The tactile stop-signal lasted for 0.5 s with a frequency of ~ 250 Hz, and about 128 μm peak-to-peak amplitude. See Fig. 1 for a depiction of the experimental setup. Upon completing both the visual and the tactile stop-signal task version, participants were escorted out of the lab and received either course credit or 10€/h as compensation. Note that participants completed both task versions in one session and the order was counterbalanced across participants. To reduce the likelihood of strategic behavior, we followed the guidelines by Verbruggen et al (2019). In short, before each performance block as well as between sessions, participants saw the instructions on screen. In addition, whenever there was a switch in stop-signal (e.g., from visual to tactile), there was a practice round. During the practice trials, participants were closely monitored by the experimenter to make sure that they adhere to the task rules. If the experimenter noticed strategic behavior or the participant expressed that they would want to wait for the stop-signal, the participant was reminded that this was against the task rules. The software used to control the experiment was PsychoPy and the analysis software was a combination of R and SPSS.

#### Stop-signal task

Each session consisted of a total of 288 trials, containing 75% go- and 25% stop-trials. The 288 trials were divided into 4 blocks with a 15 s break in between blocks. Participants were instructed to react as fast and accurately as possible to the go-stimulus (i.e., a white arrow on black background) with the left or right arrow key and withhold their reaction when a stop-signal (i.e., a vibration on the left hand or a color change of the arrow) occurs. Reactions should occur with the index and middle finger of the right hand. The go-stimulus was presented for a maximum of 1500 ms or until reaction. The stop-signal was either presented as a visual color change (from white to blue) or a tactile stimulation of the left hand following a variable delay (the Stop-Signal Delay, SSD). The SSD represents the delay between the onset of the go- and the stop-signal and was initially set to 250 ms. The SSD was continuously adjusted with the staircase procedure to obtain a probability of responding of 50%. After the reaction was successfully stopped (i.e., button press was inhibited), the SSD was increased by 50 ms, whereas when the participants did not stop successfully, the SSD was decreased by 50 ms. Several different performance measures were logged and calculated including the SSD and the probability of making a (wrong) response when a stop-signal is presented (*p*(*response|signal*)). Furthermore, two variables that are directly related to accuracy were logged: first, the amount of *omission errors* (reflecting the probability of missed response on go-trials) and, second, the *choice errors* (reflecting the probability of a wrong response on go-trials). Additionally, we logged two RT variables; *go-RT* reflects the speed of correct responses on trials without a stop-signal, and *stop-failure RT*, which indicates the latency of the incorrectly executed response on stop-signal trials. Furthermore, the probability of a *correct inhibition* (i.e., the likelihood of inhibiting an already initiated action) was recorded for each participant. Most importantly, the stop-signal reaction time (SSRT) could be calculated based on a participant’s performance. The estimation of the SSRT was based on the integration method with replacement of omissions. Broadly speaking, SSRT represents the latency of the stop-process and is calculated by subtracting the SSD from the Go-Reaction Time (for a discussion on SSRT calculation methods, see Verbruggen et al. 2019). Note that although SSRT is dependent on SSD, they are not interchangeable with regards to their interpretation. SSRT reflects the unobservable, average duration of the reactive inhibition process, while SSD is indicative of the time needed to reach the point of no-return after which an action cannot be inhibited anymore in 50% of cases.

#### Analysis plan

We followed the recommendations by Verbruggen and colleagues (Logan 2015; Verbruggen et al. 2019). First, we tested the horse-race assumption for every participant by comparing signal-response reaction time (RT) and go-RT. The horse-race assumption states that SSRT can only reliably be estimated if the RT on unsuccessful stop-trials is smaller than the mean go-RT. Second, participants were excluded if their p(response|signal) was smaller than 0.25 or larger than 0.75 in either session. Third, outliers were determined based on the Tukey outlier criterion (Tukey 1977), and removed if the accuracy of go-trials was 3 or more standard deviations below the sample. Based on these criteria, only one participant needed to be excluded, resulting in a final sample of 23 participants. All performance variables were submitted to a 2 (stop-signal modality: visual vs. tactile) × 2 (order: visual first vs. tactile first) repeated-measures ANOVA.

#### Transparency and openness

All studies within this manuscript are compliant with the Transparency and Openness (TOP) guidelines. As such all relevant data is available online (see authors note at the end of the manuscript). All data within this manuscript were collected at the University of Trier (Germany) in 2021 and 2022. Although given that the participants stem predominantly from a student population and given the place of data acquisition, the findings are unlikely to be strongly influenced by the sample. This is because the underlying process of reactive response inhibition should be culturally unspecific and universal to human behavior. However, it is unclear whether or not a significantly older or younger population may react differently and therefore yield different results.

### Results

**Fig. 2:**
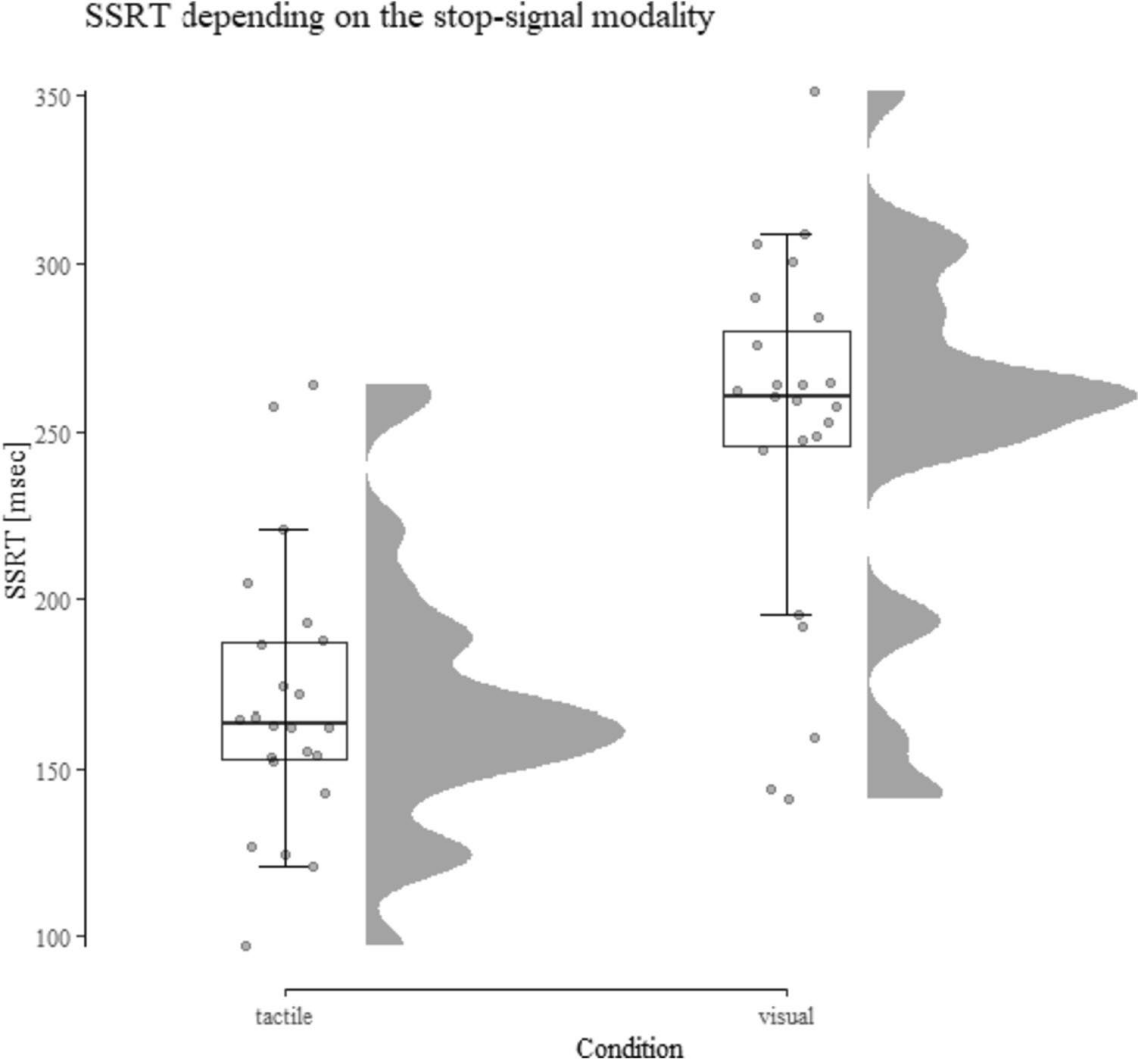
SSRT depending on the stop-signal modality and task condition (Allen et al. 2019). Results show significantly shorter SSRTs (i.e., a performance increase) in the tactile condition compared to the visual condition

Table 1 displays the performance data for study 1 and Fig. 2 shows the key result.

**Table 1:** Descriptive performance data from Study 1 depending on the condition (tactile vs. visual stop-signal)

	Tactile stop-signal	Visual stop-signal
SSRT	158 ± 42	261 ± 40
SSD	471 ± 211	357 ± 185
Correct Go-RT	660 ± 201	651 ± 186
Overall accuracy	99.44 ± 0.68	99.15 ± 0.92

Standard deviations shown in parenthesis. SSRT, SSD, and Correct Go-RT are shown in milliseconds and overall accuracy in percent

#### Preliminary SST analysis

To validate the gathered data, it is recommended to show that there is a statistical difference between the average stop-failure RT (i.e., RTs of false responses during stop-trials) and the average go-RT (i.e., RTs of correct responses during go-trials) for each experimental condition (Verbruggen et al. 2019; Verbruggen and Logan 2015). We crossed trial type (signal vs. go), order (visual first vs. tactile first), and stop-signal modality condition (visual vs. tactile) in a 2 × 2 × 2 ANOVA. As expected, results show significantly different RTs between signal (i.e., wrongful responses during stop-trials) and go (i.e., correct go responses during go-trials) trials as indicated by the significant main effect of trial type, *F*(1, 21) = 66.56, *p* < 0.001, *η*_*p*_^2^ = 0.76). Specifically, the stop-failure RTs were faster as compared to the go-RTs. No other significant effect emerged.

#### Stop-signal reaction time

The 2 (stop-signal modality: visual vs. tactile) × 2 (order: visual first vs. tactile first) ANOVA revealed a significant effect of modality *F*(1, 21) = 101.97, *p* < 0.001, *η*_*p*_^2^ = 0.83. SSRT in the tactile condition was significantly faster compared to the visual condition (158 ms vs. 261 ms), indicating more efficient inhibitory control in the tactile condition. There was no main effect of order and no interaction of order and condition (both *F* < 1 and *η*_*p*_^2^ < 0.0001).

#### Stop-signal delay

Analysis showed a main effect of condition [*F*(1, 21) = 65.14, *p* < 0.001, *η*_*p*_^2^ = 0.76] but no main effect of order (*F* < 1 and *η*_*p*_^2^ = 0.04). This is reflective of the SSD being, on average, higher in the tactile condition compared to the visual condition (471 ms vs. 357 ms), which is also indicative of a better reactive response inhibition in the tactile condition compared to the visual one. Further, the two-way interaction reached statistical significance, *F*(1, 21) = 23.20, *p* < 0.001, *η*_*p*_^2^ = 0.56. This interaction shows a time-effect from session 1 to 2. Specifically, in the visual first (i.e., visual–tactile as stop-signal order) condition, SSD increases from 352 to 534 ms, and in the reversed order, SSD changed from 396 to 362 ms.

#### Correct go-RT

We did not find significant main effects of order [*F*(1, 21) = 1.35, *p* = 0.26, *η*_*p*_^2^ = 0.06] or condition (*F* < 1 and *η*_*p*_^2^ = 0.013). However, the two-way interaction was significant [*F*(1, 21) = 11.51, *p* < 0.01, *η*_*p*_^2^ = 0.35]. The interaction is reflective of a time-based effect, as, in both conditions, the Go-RT increased from session 1 to 2. In detail, Go-RT increases from 672 to 727 ms in the visual first (i.e., visual–tactile as stop-signal order) order condition, and from 588 to 628 ms in the tactile–visual condition.

#### Error rates

With regards to performance errors, due to the overall low number of errors, omission and commission errors were combined and overall accuracy was submitted to the analysis. Neither the main effect of order [*F*(1, 21) = 1.88, *p* = 0.19, *η*_*p*_^2^ = 0.08], nor the main effect of condition (*F* < 1 and *η*_*p*_^2^ = 0.045) or the interaction (*F* < 1 and *η*_*p*_^2^ < 0.0001) reached statistical significance.

### Discussion

Overall, the results reveal that inhibition in a task with visual go-stimuli is more effective when the stop-signal is tactile as compared to a visual stop-signal. This is evidenced by significantly lower SSRT (and higher SSD) in the tactile condition. However, the current experimental setup entails a potentially problematic confounding variable: the stop-signal location. Specifically, the visual stop-signal is presented at the same location as the go-signal, while the tactile stop-signal is presented on the participants’ hand and not linked to the go-signal.

It should be noted that in study 1, the RTs generally increased from session 1 to session 2; regardless of the order and thus independent of the stop-signal type. This performance change may be to general fatigue after having already performed a somewhat demanding task. However, the effect did not interact with the condition order (i.e., with the order of the stop-signal variants that were encountered) and thus most likely did not affect the overall main result reported here. Alternatively, it is possible that the change in stop-signal modality condition led the participants to require an adjustment period which led to longer RTs. However, given that each participant had ample time to practice, this also seems unlikely.

In this study, the order in which participants encountered the stop-signal conditions influenced their performance. Specifically, if participants were first confronted with a tactile stop-signal, their correct go-RTs were generally faster across both sessions as opposed to encountering the visual stop-signal first. Either, this may be due to the fact that the cohort of individuals that was sorted into the respective order condition was just generally different in response speed and the overall sample size was not sufficient to equalize this effect between “groups”. Or the tactile stop-signal led to generally higher performance and this effect carried over to the second session; potentially because participants subjectively experienced their performance as better or because they enjoyed the task more. However, please note that such a starting difference in a primarily within-subject design should not affect the main outcome as the main analysis focuses on differences between conditions within each subject.

## Study 2: investigating the effect of spatial separation

The second study utilized a slightly different experimental setup in relation to how the stop-signals are presented. In short, the stop-signals were presented using a multisensory cube through which visual as well as tactile stimuli can be presented (for a detailed description of the multisensory cube, see Merz et al. 2019). This enabled us to present both the visual and tactile stop-signals at the same location and not connected to the location of the go-signal. We hypothesized that the tactile condition would yield comparable results as in Study 1, as the two conditions are essentially identical apart from the specific instruments used to elicit the tactile stimulation. With regards to the visual condition, three outcomes were possible. First, the location of the visual stop-signal has no influence on performance. Second, performance could be increased if the visual stop-signal is presented at the same location as the go-stimulus, as compared to the stop-signal being presented at a different location. This may be due to attention already being focussed on this location and the stop-signal popping out of the environment (Turatto and Galfano 2000). Third, performance in the peripheral stop-signal condition may be increased due to an effect akin to inhibition of return (IOR) (Klein 2000). IOR effects can be observed when a stimulus appears at a previously cued location and result in longer RTs to that stimulus. Typically, IOR effects occur when the stimulus is presented 200–300 ms after the cue. Notably, in the SST, the stop-signal appears at a specified delay after the go-signal; usually on average between 200 and 500 ms (see, for example, Friehs et al. 2020a, b; Friehs and Frings 2018, 2019), which corresponds precisely to the time frame in which the IOR effect can be observed.

### Method

#### Sample

The power calculation and sample size did not change from study 1. Consequently, we collected 24 participants (24 female, aged 19–30) who did not participate in study 1. Participants had a mean age of 22.50 (SD = 2.59). All but two people were right-handed.

#### Design

See study 1.

#### Stop-signal task

**Fig. 3:**
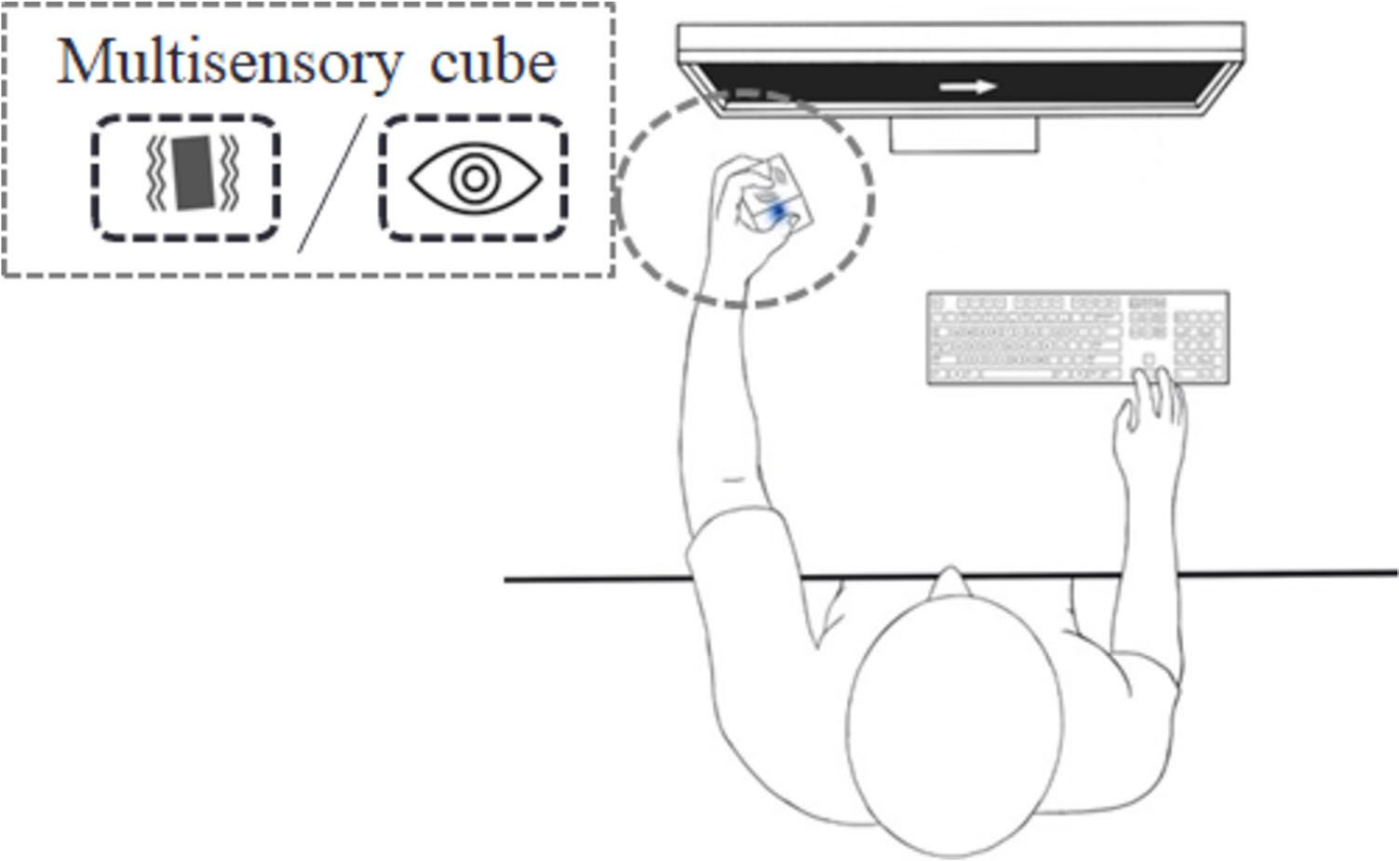
Experimental setup for study 2. Both the tactile and visual stop-signals are presented peripherally and spatially separated from the go-signal on screen

The SST was identical to study 1. The only difference was how the presentations of the stop-signals were handled. Here, we used a multisensory cube with two LEDs and two tactors to present the stop-signal (for details, see Jensen et al. 2020; Merz et al. 2019). Briefly, the stimuli were presented via a custom-made cube with two LEDs (5 mm^2^ each) and two factors. Each tractor is connected to a vibration damper to ensure that vibrations are only felt at the intended location. The cube is 70 mm^3^ with a total weight of approximately 125 g which ensures comfortable handling for the whole duration of the experiment. For a technical description including a 3D rendering of the cube, we refer the reader to the appendix of Merz et al. (2019) Participants held this device in their left hand. See Fig. 3 for a visualization. The stop-signals were akin to those in study 1. To reiterate: the tactile stop-signal lasted for 0.5 s with a frequency of ~ 250 Hz, and about 128 μm peak-to-peak amplitude. The visual stop-signal was matched with regards to duration and onset timing.

#### Analysis plan

The analysis procedure was identical to study 1 with the addition of cross-experimental comparisons of the performance indicators. Based on the performance criteria, 2 participants had to be removed, resulting in a final sample of *N* = 22 participants.

### Results

**Fig. 4:**
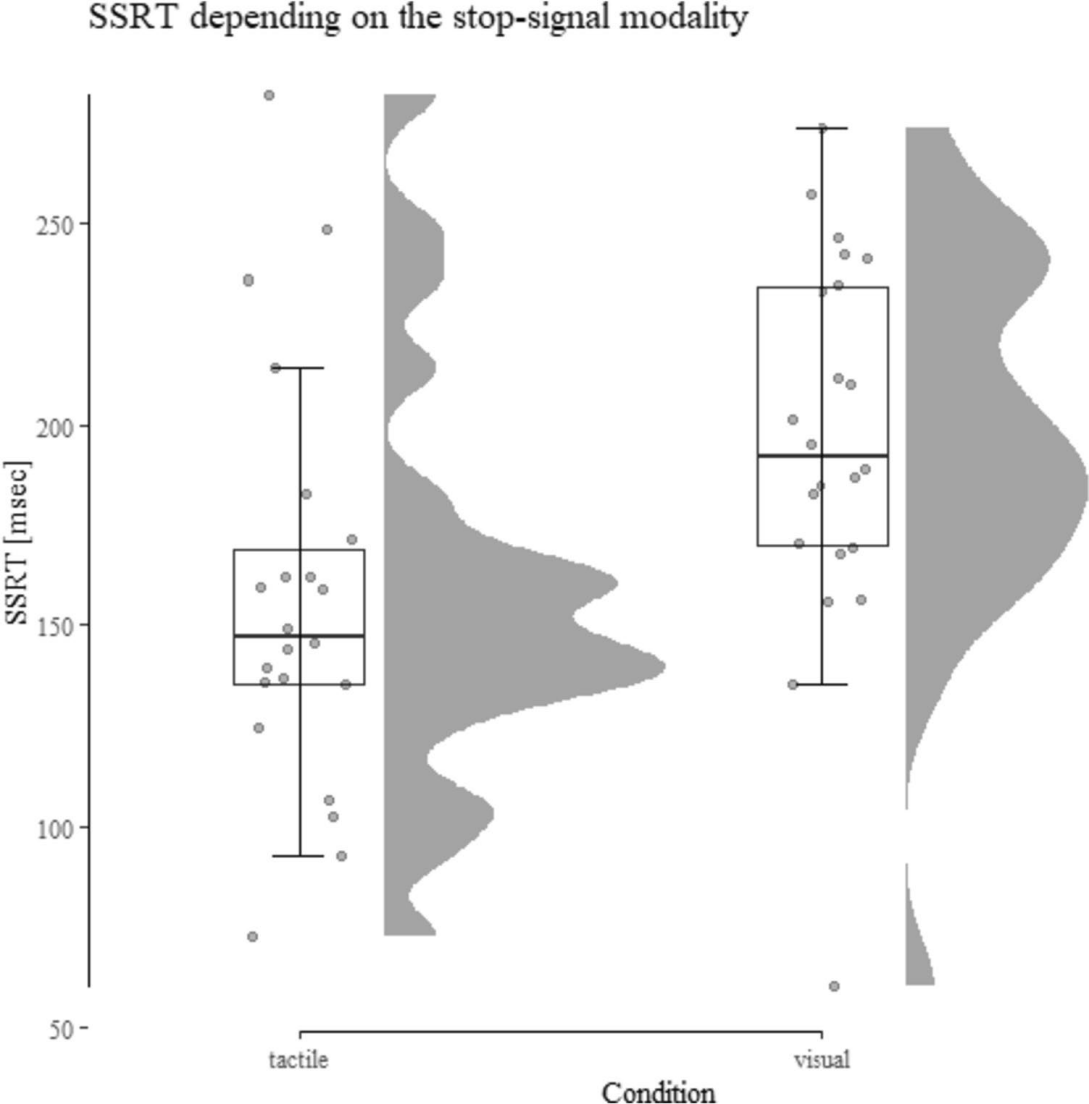
SSRT depending upon the stop-signal modality and task condition. Results show a significant decrease in SSRT (i.e., a performance increase) in the tactile condition compared to the visual condition

**Fig. 5:**
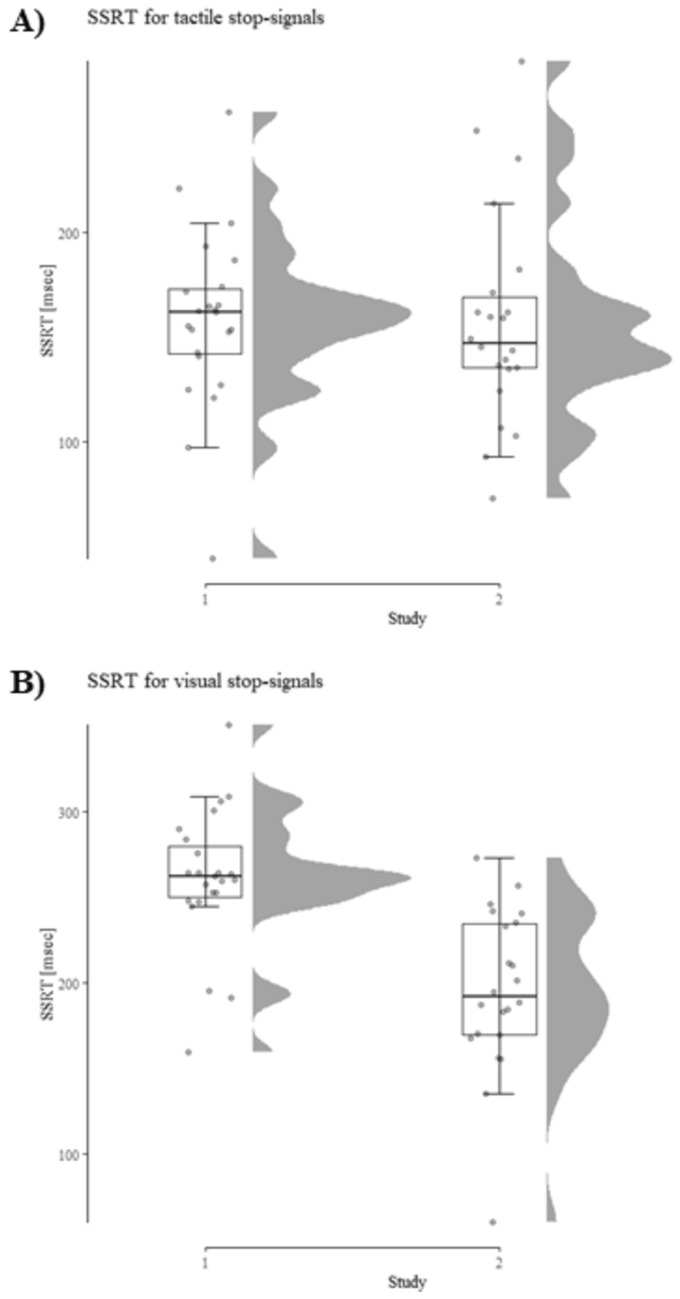
**A** SSRT for tactile stop-signals in study 1 and 2. Performance was virtually identical across both studies (mean SSRT 158 ms vs. 157 ms). **B** SSRT for visual stop-signals in study 1 and 2. Untangling the visual stop-signal from the visual go-signal improves performance, as indicated by a significant reduction in SSRT from study 1 to 2

Performance data are presented in Table 2 and SSRT depending on the condition is displayed in Figs. 4 and 5.

**Table 2:** Descriptive performance data from Study 2 depending on the condition (tactile vs. visual stop-signal)

	Tactile stop-signal	Visual stop-signal
SSRT	157 ± 51	196 ± 48
SSD	479 ± 259	434 ± 227
Correct Go-RT	670 ± 251	660 ± 227
Overall accuracy	99.35 ± 0.74	99.20 ± 0.95

Standard deviations are shown in parenthesis. SSRT, SSD, and Correct Go-RT are shown in milliseconds and overall accuracy in percent

#### Preliminary SST analysis

Data validation followed the same principles as explained in Study 1. Results revealed a significant main effect of trial type *F*(1, 20) = 70.48, *p* < 0.001, *η*_*p*_^2^ = 0.78, which satisfied the analysis prerequisite.

#### SSRT

The ANOVA revealed a significant effect of condition *F*(1, 20) = 19.04, *p* < 0.001, *η*_*p*_^2^ = 0.49. SSRT in the tactile condition was significantly faster compared to the visual condition (157 ms vs. 196 ms), indicating more efficient inhibitory control in the tactile condition. There was no main effect of order [*F*(1, 20) = 1.31, *p* = 0.27, *η*_*p*_^2^ = 0.061] and no interaction of order and condition (*F* < 1 and *η*_*p*_^2^ = 0.012).

#### SSD

There was no main effect of condition [*F*(1, 20) = 3.22, *p* = 0.07, *η*_*p*_^2^ = 0.156] and no interaction of order × condition (*F* < 1 and *η*_*p*_^2^ = 0.032). Further, analysis revealed a significant main effect of order [*F*(1, 20) = 6.22, *p* < 0.05,* η*_*p*_^2^ = 0.24]. Specifically, SSD was larger overall if the visual condition came first as compared to the other order (569 ms vs. 344 ms).

#### Correct go-RT

Neither the main effect of condition nor the interaction did reach statistical significance (both *F’s* < 1 with *η*_*p*_^2^ = 0.009 and *η*_*p*_^2^ = 0.20, respectively). However, the main effect of order was significant [*F*(1, 20) = 5.76, *p* < 0.05, *η*_*p*_^2^ = 0.22]. Generally, the RT was larger if the visual condition came first (773 ms vs 557 ms).

#### Error rates

Neither the main effect of condition, nor the main effect of order or the two-way interaction was significant (*F’s* < 1).

#### Cross-experimental comparison

To test whether the data pattern from both studies is robust, we combined both datasets and computed a 2 (condition: tactile vs. visual) × 2 (study: study 1 vs. study 2) repeated-measures ANOVA. For SSRT, a significant effect of condition emerged [*F*(1, 43) = 114.22,* p* < 0.001,* η*_*p*_^2^ = 0.73] as well as a significant main effect of study [*F*(1, 43) = 7.89,* p* < 0.01,* η*_*p*_^2^ = 0.16]. Thus, when faced with the tactile condition, participants were able to reach the point-of-no-return later, and had a better response inhibition as compared to the visual condition. Further, generally, SSRT was larger in study 1 as compared to study 2. However, the main effect of study is driven by the difference between the visual conditions as indicated by the significant interaction [*F*(1, 43) = 23.91,* p* < 0.001,* η*_*p*_^2^ = 0.36] and post hoc comparisons. A comparison between SSRTs with a visual stop-signal yielded a significant result [*t*(43) = 4.98*, p* < 0.001, *Cohens d* = 1.49]. This indicates an improvement from study 1 to study 2 (261 ms vs. 196 ms) and supports the hypothesis that a location decoupling of the stop- and go-signal improve SSRT. There was no difference between the tactile conditions (157 ms vs. 158 ms). For a visualization, see Fig. 5.

### Discussion

The results of the second study reveal an advantage of a tactile stop-signal compared to the visual stop-signal. This result is in line with Study 1 and notably the SSRT in the tactile condition is descriptively almost identical across experiments (157 ms vs. 158 ms). Further, when the visual stop-signal conditions were compared across experiments, the results revealed that decoupling the stop-signal and the go-signal with regards to their location improves performance (see Fig. 5). Put differently, if the stop-signal is presented at a different location than the go-signal, the overall reactive inhibition performance improves. Thus, in sum, the results of Study 2 may be explained by an advantageous modality effect for the tactile modality and a location effect for the visual modality. It may be that participants partially re-direct their attention towards the location of the stop-signal, but given that all general go-RTs as well as error rates are comparable across conditions and experiments, this explanation cannot fully account for the results, although more eye-movement may have occurred (see also Verbruggen et al. 2014). Further, evidence by Friehs et al. (2020b) suggests that even in a more visually complex environment, which modified gaze and eye movements, the performance is comparable to a standard SST version. However, in the present study, it remains unclear whether these effects are robust against distractions and also can be observed in visually noisy environments. Understanding signal processing in distracting environments is especially important since everyday life is seldomly without distractors. Thus, in study 3, we aim to investigate the effect of visual distractions, which are ubiquitous in everyday life (e.g., imagine a busy road when driving a car). Note that in contrast to study 1, two of the subjects were left-handed and the sample was thus not completely right-handed. Since the task required subjects to react with their right hand, their performance could have potentially been slower. However, this was not reflected in the data. There is recent evidence that handedness does not impact general performance in the SST, and since we employed a within-subjects design, the impact of a general RT increase on comparisons across conditions should be negligible (Mancini and Mirabella, 2021).

## Study 3: response inhibition in visually complex environments

The third study investigated the effect of visual distractors on SST performance with a tactile or visual stop-signal. In the visual distraction literature, it is well known that the presence of task-irrelevant stimuli affects performance: If irrelevant stimuli are present, reaction times are increased compared to environments free of other, irrelevant stimuli (Pratt and Abrams 1994). This effect is further amplified if the irrelevant stimuli become intrusive (e.g., Theeuwes 1992) or use the same feature dimensions as task-relevant stimuli (Found and Müller 1996; Memelink and Hommel 2013). Specifically, we wondered how adding visual distractors to our displays affected task performance in general and if the modality the distractor is presented in affects our task differently, depending on the modality that is task-relevant. Importantly, both the tactile and the visual stop-signal were presented peripherally, as in Study 2. We hypothesized that the tactile stop-signal condition would yield similar results as compared to the previous two studies. If the prediction that tactile stop-signals retain their performance facilitating effect even under distracting conditions, this has important implications for the technological development of response signals (e.g., warning signals in cars or human–machine-interactions in general). With that being said, for the visual stop-signal condition, two results were possible: Either the performance advantage observed in Study 2 could be translated to visually noisy environments, or the performance in the visual stop-signal condition would suffer due to the visual distractors in Study 3.

### Method

#### Sample

We collected 24 new participants (14 female, 10 male, aged 20–40) with a mean age of 24.17 (SD = 4.66). One participant was left-handed.

#### Design

See study 1 and 2.

#### Stop-signal task

**Fig. 6:**
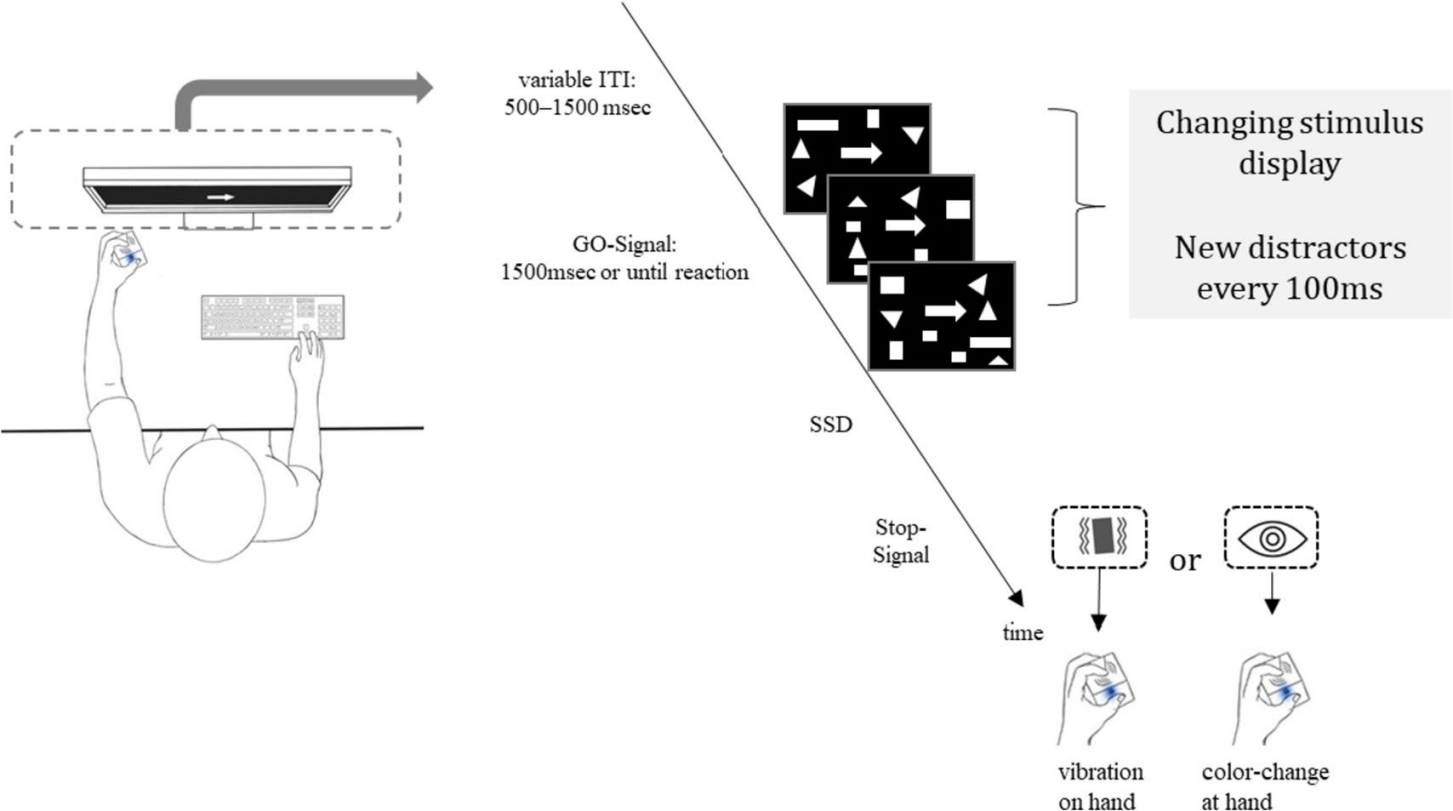
Experimental setup of study 3. Visualization of the stimulus display and the participant setup in the laboratory

The SST was identical to study 2. The only difference was that visual distractors were presented alongside the go-signal. The shapes that made up the arrow used as the go-signal (i.e., triangles and rectangles) changed position randomly on the screen every 100 ms starting with the go-signal onset. In detail, 20 distractors appeared and every 100 ms, and the old distractors were replaced by 20 new distractors at new random locations to ensure a perceptual load during the whole trial; this was required, because the delay between the go-stimulus and the stop-signal varied (for a similar approach see Verbruggen et al. 2014). See Fig. 6 for a visualization.

#### Analysis plan

The analysis procedure was identical to study 1 and 2 with the addition of cross-experimental comparisons of the performance indicators. Based on the performance criteria, one participant had to be removed resulting in a final sample of *N* = 23 participants.

### Results

**Fig. 7:**
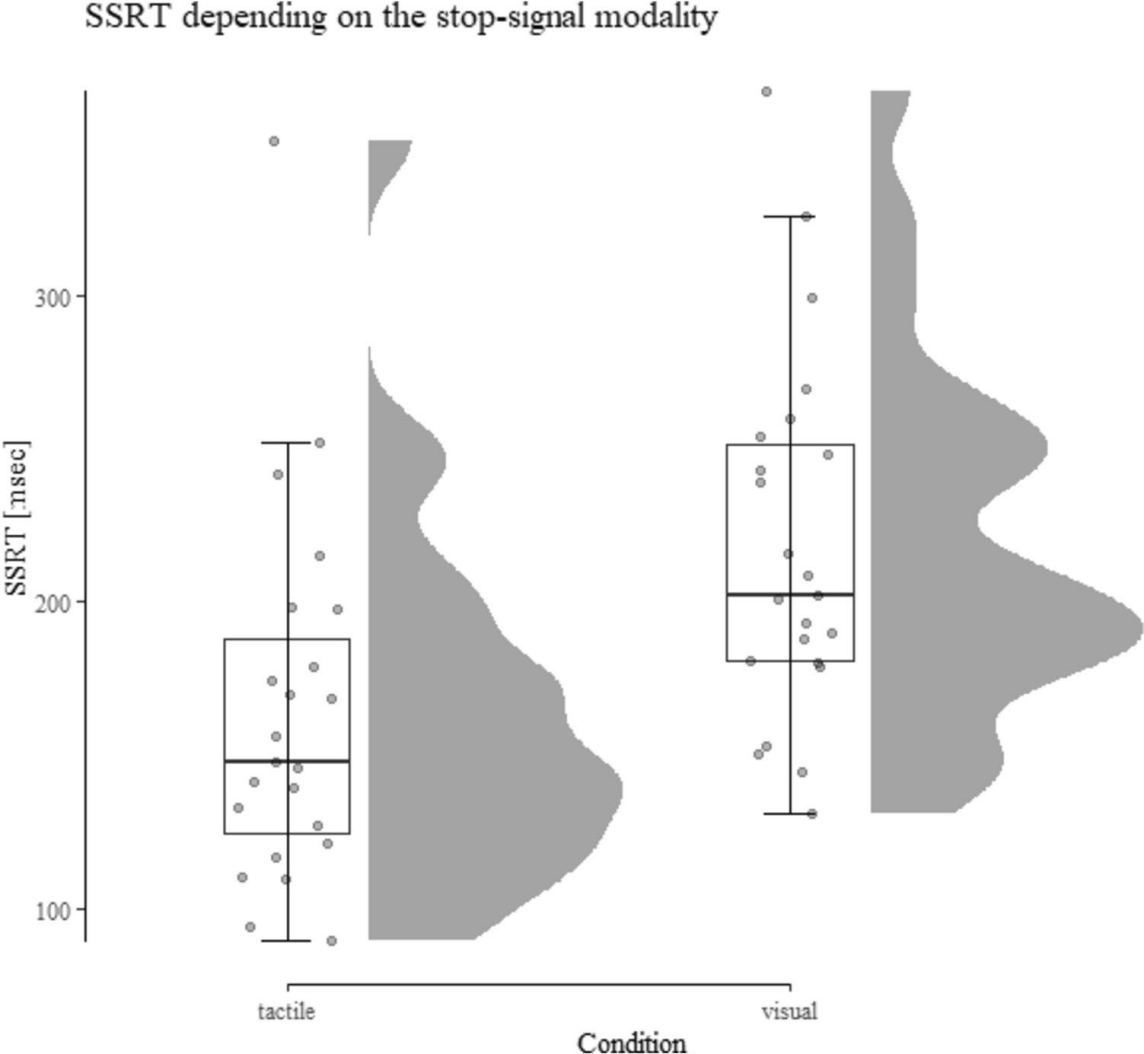
SSRT depending on the stop-signal modality and task condition. Results show a significant decrease in SSRT (i.e., a performance increase) in the tactile condition compared to the visual condition

**Fig. 8:**
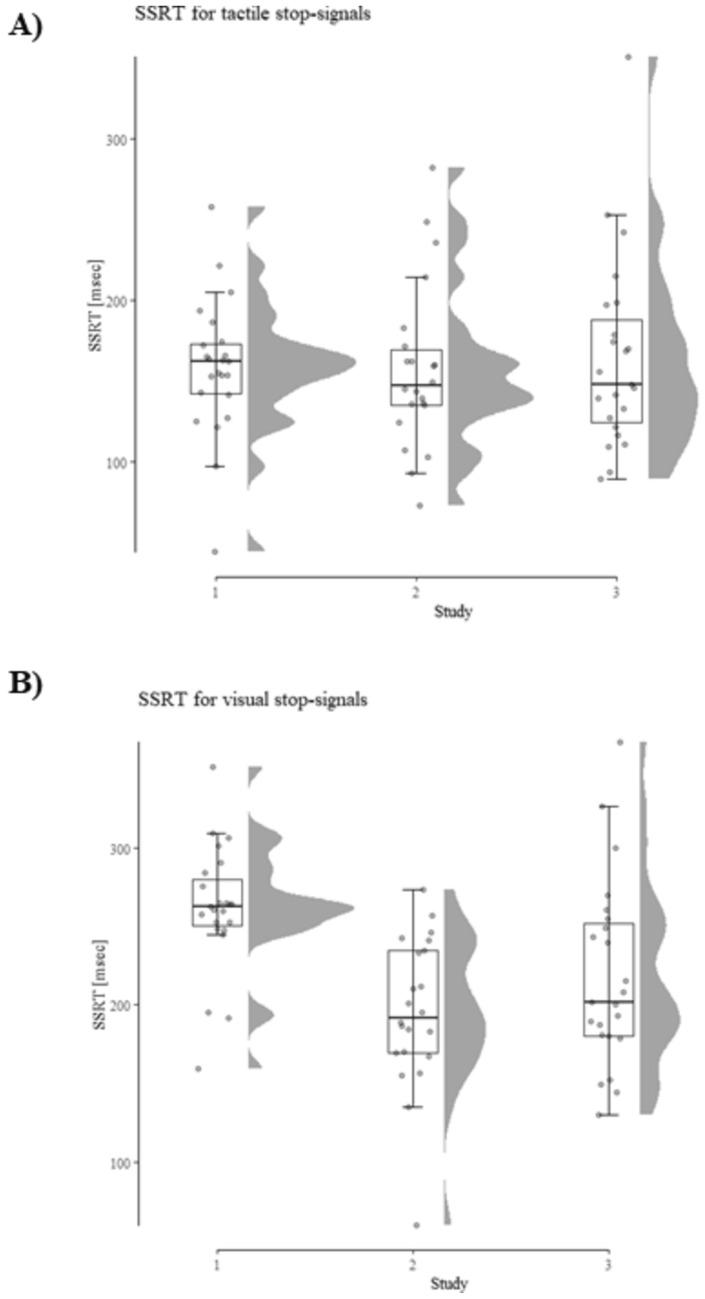
**A** SSRT for tactile stop-signals in Study 1, 2, and 3. Performance was virtually identical across all three studies. **B** SSRT for visual stop-signals in Study 1, 2, and 3. Untangling the visual stop-signal from the visual go-signal improves performance, as indicated by a significant reduction in SSRT from Study 1 to 2. Further, this advantage remains when visual distractors are added. Note that although descriptively, the distributions and variances differ across conditions and studies, there was no not significant difference. Levene tests yield insignificant results, for both tactile [*F*(2,65) = 1.11, *p* = 0.38 for the estimation based on the mean and *F*(2,65) = 0.80, *p* = 0.46 for an estimation based on the median] and visual [*F*(2,65) = 2.49, *p* = 0.09 for the estimation based on the mean and *F*(2,65) = 1.83, *p* = 0.18 for an estimation based on the median]. This indicates comparable population variances and renders the *F*-tests interpretable

Performance data are presented in Table 3 and SSRT depending on the condition is displayed in Figs. 7 and 8.

**Table 3:** Descriptive performance data from Study 3 depending on the condition (tactile vs. visual stop-signal)

	Tactile stop-signal	Visual stop-signal
SSRT	164 ± 60	218 ± 59
SSD	526 ± 241	487 ± 234
Correct Go-RT	716 ± 244	742 ± 233
Overall accuracy	0.99 ± 0.02	0.99 ± 0.02

Standard deviations shown in parenthesis. SSRT, SSD, and Correct Go-RT are shown in milliseconds and overall accuracy in percent

#### Preliminary SST analysis

Data validation followed the same principles as explained in Study 1 and 2. Results revealed a significant main effect of trial type *F*(1, 21) = 73.65, *p* < 0.001, *η*_*p*_^2^ = 0.78, which satisfied the analysis prerequisite.

#### SSRT

The ANOVA revealed a significant effect of condition *F*(1, 21) = 15.72, *p* < 0.001, *η*_*p*_^2^ = 0.43. SSRT in the tactile condition was significantly lower compared to the visual condition (164 ms vs. 218 ms), indicating more efficient inhibitory control in the tactile condition. There was no main effect of order and no interaction of order and condition (both *F’s* < 1 with *η*_*p*_^2^ = 0.032 for the main effect order and *η*_*p*_^2^ < 0.0001 for the interaction effect).

#### SSD

There was no main effect of condition [*F*(1, 21) = 2.87, *p* = 0.11, *η*_*p*_^2^ = 0.12] or order [*F*(1, 21) = 1.74, *p* = 0.20, *η*_*p*_^2^ = 0.076] and no interaction of order × condition [*F*(1, 21) = 2.6, *p* = 0.12, *η*_*p*_^2^ = 0.11].

#### Correct go-RT

Neither the main effect of condition [*F*(1, 21) = 1.09, *p* = 0.31, *η*_*p*_^2^ = 0.049], nor the main effect of order [*F*(1, 21) = 2.0, *p* = 0.19, *η*_*p*_^2^ = 0.086] or the two-way interaction was significant [*F*(1, 21) = 1.87, *p* = 0.19, *η*_*p*_^2^ = 0.082].

#### Error rates

Neither the main effect of condition (*F* < 1 and *η*_*p*_^2^ = 0.004), nor the main effect of order [*F*(1, 21) = 1.99, *p* = 0.17, *η*_*p*_^2^ = 0.087] was significant. The two-way interaction reached significance [*F*(1, 21) = 7.78, *p* < 0.05, *η*_*p*_^2^ = 0.27]. This interaction indicates that in the visual condition, participants made similar amounts of errors regardless of the order, but in the tactile condition, participants’ people made less errors when that condition came first.

#### Cross-experimental comparison

We compared data from studies 2 and 3 in a 2 (condition: tactile vs. visual) × 2 (study: study 2 vs. study 3) repeated-measures ANOVA. Most importantly, for SSRT, a significant main effect of condition emerged only for SSRT [*F*(1, 43) = 33.22,* p* < 0.001*, η*_*p*_^2^ = 0.47]. Thus, when faced with the tactile condition, participants were able to reach the point-of-no-return later, and had a better response inhibition as compared to the visual condition. Further, there was no main effect of study [*F*(1, 43) = 1.09,* p* = 0.30,* η*_*p*_^2^ = 0.03], nor an interaction between the two factors (*F* < 1). When the data from all three studies were pooled, the main effect of condition remained significant [*F*(1, 66) = 107.28,* p* < 0.001*, η*_*p*_^2^ = 0.62]. Further, a comparison between SSRTs with a visual stop-signal yielded a significant main effect of study [*F*(2, 65) = 7.93, *p* < 0.001, *η*_*p*_^2^ = 0.20]. In addition to the above-described significant difference between visual SSRTs for Study 1 and 2, SSRTs were also different when Study 1 and 3 were compared (*p* < 0.05). However, the comparison of Study 2 and 3 yielded no significant difference (*p* = 0.31). For a visual representation, see Fig. 8.

#### Non-parametric analysis

To validate our results and because all studies had outliers in their datasets, non-parametric tests were carried out. First, a Kruskal–Wallis test across studies revealed equal distributions across studies for SSRTs in the tactile condition (*p* = 0.76), but unequal distributions for SSRTs in the visual conditions (*p* < 0.001). Further, overall (*Z* = −6.50, *p* < 0.001) as well as in studies 1 (*Z* = −4.20, *p* < 0.001), 2 (*Z* = −3.50, *p* < 0.001), and 3 (*Z* = −3.22, *p* < 0.001) the Wilcoxon Signed-Rank test revealed a significant difference between visual and tactile SSRT.

### Discussion

Results show that the effects of the two previous studies can be translated to visually noisy environments, which is evidenced by an advantage of tactile stop-signals compared to visual stop-signals. Further, the advantage of disentangling the location of go- and stop-signal remained unchanged for visual stop-signals. Put differently, visual noise does not significantly impact reactive response inhibition if the visual stop-signal is presented at a different location as the go-signal and the visual noise.

## General discussion

In three separate experiments, this study investigated the impact of cross-modal stop-signals on response inhibition. Overall, the results show that a tactile stop-signal leads to a more efficient reactive response inhibition process compared to a visual stop-signal. Specifically, Study 1 shows that a tactile stop-signal leads to better performance compared to a visual stop-signal. However, the stop-signal modality and the stop-signal location were confounded in the experimental setup. Study 2 reveals a continued advantage of the tactile stop-signal even if the visual stop-signal is also disentangled from the location of the visual go-signal. Further, cross-experimental comparisons evidence faster inhibition in response to visual stop-signals in Study 2 compared to Study 1, which shows that differentiating the location of stop- and go-signals leads to faster inhibition. The results of Study 3 replicate and extend the previous results and demonstrate a continued advantage of the tactile stop-signals even when visual distractors are added to the task. Furthermore, results indicate that responses to visual stop-signals are more robust when the stop-signal is presented at a different location than the go-signal as well as the distractors.

The combined results from three studies provide deeper insights into the basis of reactive response inhibition. It should be noted that at first glance, these results may not be in line with the study by Verbruggen et al. (2014). Their data show worse performance in response to peripheral visual stop-signals. However, the stop-signal they used was a bold frame around the display screen, which is less noticeable compared to a blinking LED presented peripherally. Thus, even though, in both studies, a peripheral stop-signal was used the ease of perception for these stimuli differs drastically. Therefore, future studies may investigate the effect of LED stop-signals in different locations by varying the distance to the go-signal continuously.

Collectively, the previous and present results provide more support for the capacity sharing account, which states that as cognitive demands increase (e.g., via selective and more complex stopping rules, low discriminability, or intensity of the stop-signal), the processing rates for individual stimuli decrease and RTs are slowed (Verbruggen and Logan 2015). Within this framework, pulling apart the location of stop- and go-signal or using a tactile stop-signal would make stopping more efficient, because stop-signals are easier to detect. This line of reasoning fits models from visual search and visual attention research. Specifically, it is assumed that stimuli are processed more effectively when a specific stimulus has a high local feature contrast and the stimulus is significant for task-goal completion (Nordfang et al. 2013; Rangelov et al. 2012; Zehetleitner et al. 2012). Thus, a tactile stop-signal in an otherwise visual task makes it easy to separate the stop-signal from the go-stimulus input. Further, the tactile input benefits from directed attention because of the task goal and its relevance to the task (i.e., stopping in response to a tactile input). A visual stop-signal in an otherwise also visual task is harder to separate from the go-stimulus unless it is presented at a different location compared to the go-signal as well as potential distractors. As an extension of these lines of reasoning, one may also speculate whether stop-signals covering multiple modalities have an additive or even multiplicative effect on stopping performance, or if there is a limit for this kind of performance enhancement. However, one might consider the possible alternative explanation, that tactile stop-signals are preferentially processed on a physiological level. However, this explanation is unlikely given that research demonstrated that touch needs to precede visual input to be perceived as synchronous (Harrar and Harris 2005, 2008; Hirsh and Sherrick 1961; Shore et al. 2006; Spence et al. 2001, 2003). Nevertheless, further experimentation is necessary to fully understand the role of stop-signal modality. This may entail systematically utilizing all modalities as either the stop- and go-signal. To support the proposed cross-modal tactile stopping benefit proposed in the present manuscript, an auditory go-signal and a tactile stop-signal experiment should lead to better performance compared to a dual-tactile signal setup.

Furthermore, understanding how response inhibition works in the general population may help us to better understand suboptimal response inhibition, as well. For example, it has been suggested that young children and older adults on average perform worse in tasks requiring response inhibition (Pauwels et al. 2019; Rush et al. 2006; Van Den Wildenberg and Van Der Molen 2004 but see also Rey-Mermet and Gade 2018). Consequently, it is an open question whether elderly people benefit from cross-modal stop-signals to the same degree that young participants do. Transferring our experiments to children and older participants could be a next step towards understanding how cognitive abilities change over the lifespan. In fact, recent evidence suggest that older adults and children can benefit from additional manipulations of classical cognitive tasks, such as the addition of affective information (Zinchenko et al. 2017, 2019). Similarly, understanding the basis of response inhibition in the healthy population could inspire new and improved interventions for psychiatric disorders, such as substance abuse, binge-eating, compulsive gambling, ADHD, and obsessive compulsive disorder; all of which correlate with reduced response inhibition (Goudriaan et al. 2006; Kirsten et al. 2022; Lijffijt et al. 2005; Lipszyc and Schachar 2010; Woolley et al. 2008).

However, from a theoretical perspective, another aspect should be considered. Although, as pointed out, SSRT has been conceptualized to measure reactive response inhibition, there is an ongoing discussion about what SSRT represents and some researchers propose alternative measures (Diesburg and Wessel 2021; Huster et al. 2021; Jana et al. 2020). In short, unless other measures such as EMG recordings are also taken into account, SSRT alone may not fully reflect the stopping process (Bissett et al. 2021; Jana et al. 2020). Another possibility of probing behavioral inhibition is by recording motor-evoked potentials in response to stop-signals and investigating reactivity with transcranial magnetic stimulation. A reduction of motor activity in response to a stop-signal may indicate the contribution of inhibition (Choo et al. 2022; Hynd et al. 2021; Skippen et al. 2019; Wessel 2018). There is evidence that specific brain areas such as the right prefrontal cortex are involved in implementing inhibitory control, but evidence from transcranial magnetic stimulation studies is mixed (for a recent null result, Friehs et al. 2023). In fact, recently, the “pause-then-cancel” model was adapted to human action stopping (Diesburg and Wessel 2021). These researchers argue that after a salient event, such as a stop-signal, attention is oriented towards the salient-related pause, which activates unspecific motor inhibition and the appropriate action can be canceled (Huster et al. 2021; Tatz et al. 2021; Wessel 2018). Thus, although SSRT is undoubtedly linked to stopping an action, it is unclear whether the measured SSRT in an SST reflects the pause-, or cancel-process, both, or a completely different process. With regards to the present study, this line of thinking would imply that future research should corroborate our results by adding direct neural or psychophysiological components to the dependent variable list.

Further, the present results should be judged against the background of work on trigger failures in stopping. In the present research, we do not account for trigger failures (i.e., failure to initiate the stopping process), and thus, it may be possible that SSRT is overestimated (Doekemeijer et al. 2021; Jana et al. 2020; Matzke et al. 2017a, b). With that being said, SSRT should be somewhat overestimated in all sub-studies and thus not affect cross-condition or cross-study comparisons significantly. However, individuals with slower SSRT (i.e., worse performance) tend to exhibit an increase in trigger failures (Choo et al. 2022), and given that some outliers were detected in the present study, it would have been interesting to investigate trigger failures. Outliers were distributed across all sub-studies and thus probably do not account for specific effects and future studies may investigate trigger failures in response to different stop-signal modalities or environments. Nevertheless, trigger failures are potentially more common in complex and noisy environments, but a salient and effective stop-signal (such as a tactile signal in visually dominant environments according to our results) may counteract this effect.

### Future work

The present results motivate future studies to explore the nature of cross-modal stopping. First, additional evidence from eye-tracking or a horizontal electrooculogram could help understanding how the location of the stop-signal affects stopping. Investigating eye-movement patterns in response to tactile and visual stimuli at different locations may offer valuable insights into the underlying processes influencing cognitive control. Second, go- and stop-signal modality should be systematically varied. For example, an intriguing avenue for exploration involves replicating Study 2 but introducing a central tactile Go stimulus accompanied by peripheral tactile and visual stop-signals. This modification would allow investigating a second cross-modal configuration, and may thereby shed light on whether the efficacy of action stopping is inherently tied to the cross-modal nature or if tactile stimuli alone suffice. Third, a critical follow-up experiment may involve manipulating the spatial location of both Go- and Stop-Signals. For instance, placing the Go signal peripherally while locating the Stop-signal centrally, and vice versa, could clarify whether the significance lies in the peripheral nature of the stop-signal or the spatial dissociation between the two signals. This design would contribute valuable insight into the spatial dynamics governing efficient stopping. Fourth, future experiments could explore reversing the modality-specific pairings of stop- and go-signals present in the current studies. Specifically, this involves testing whether visual stop-signals coupled with tactile go-signals confer any advantages or disadvantages. This reversal would help ascertain the generalizability of the observed effects and whether tactile stimuli consistently hold an advantage in action stopping.

### Practical implications

To reiterate, two key results emerged in the present study. First, tactile warning signals lead to a more efficient response inhibition in an otherwise visual task. This is especially interesting as tactile warning signals have also shown advantageous response execution performances (e.g., a breaking response, Meng and Spence 2015), supporting the notion that the presentation of relevant action initiation as well as inhibition in touch is relevant for fast and successful behavior. Second, disentangling the location of stop- and go-signals leads to a performance increase. Warning signals and response stopping is paramount in many situations. For example, it seems important for a car manufacturer to know how to best convey a stop-signal to the driver. Certain manufacturers have already experimented with vibrating steering wheels to convey signals and some research suggests that vibrations in the seat or seat belt may also effectively draw attention (Meng and Spence 2015). Future research may specifically investigate response stopping in such environments. However, driving and stopping a car are even more complex than just inhibiting an already initiated action in a laboratory environment: to avoid hitting an object on the road, the driver either needs to reorient the car or push down the break (cf. stop-change paradigms, Verbruggen and Logan 2009). Thus, the stop-signal paradigm itself needs to be adapted to a new environment with the addition of a foot pedal to simulate a break and response control would need to be constantly tracked on the steering wheel (Morein-Zamir et al. 2006). In fact, some research groups already investigated foot-based inhibitory control (Lenné et al. 2011; Petraconi et al. 2019) and provided evidence that their neural correlates overlap (Tabu et al. 2012). Thus, some of the results of the present studies may potentially transfer to foot-based inhibitory control (i.e., a tactile stop-signal may aid in stopping a foot-response as well). Although improving driving and stopping a car may be one of the more striking applications of the present research, another potential avenue is the application of these results to improve gaming experience and performance in video games. For example, a player in a game might have to stop advancing towards the enemy, because a trap was spotted or the game may signal the player that his avatar in game has taken a hit and that the player needs to stop advancing (for a similar line of reasoning, see Friehs et al. 2020a, b). Reacting to these kinds of information may be easier and more effective when appropriate stop-signals are used. This may mean presenting stop-signals close to the avatar the player identifies with (Friehs et al. 2022a, b) or using a vibrating controller to convey the stop-signal. Increased performance can also be viewed through a different lens: making performance more consistent. More predictable human behavior may provide an autonomous system with an improved ability to anticipate a user’s behavior and in research fewer trials would be needed to accurately evaluate performance. One previously discussed way to increase performance and the consistency of behavior is to add game elements to a task to enhance the motivation to perform (Friehs et al. 2022a, b; Gallagher et al. 2023; Thirkettle et al. 2018; Wiley et al. 2021).

### Limitations

Our study has a number of limitations and some questions remain open. First, the different sub-studies focussed on the visual and tactile modality, while auditory stimuli were not used. Thus future research may extend the present results to other cross-modal task setups. Consequently, if the present results were replicated in another domain, it would strengthen the conclusions that can be drawn from this study. Thus, is may be reasonable to assume that, for example, an auditory stop-signal, compared to a visual one, should lead to more effective reactive response inhibition, especially in visually complex environments. However, the questions remains whether or not tactile and visual stop-signals yield comparable results or if tactile signals would remain superior. Second, we used a visual task as the default task version, but a primarily auditory or tactile choice-reaction task would be equally suitable. In theory, the modality of the go-signal should not matter but future research needs to confirm this assumption. Third, participants in the present studies were limited to one-handed reactions, but hypothetically, these effects should hold when effectors were changed towards reacting with foot pedals. Incidentally, a reaction with a foot pedal would be even closer to the aforementioned example of driving a car. This should be explored in future research. Fourth, while study 1 utilized a color changing stop-signal on screen, studies 2 and 3 used a blinking LED light from a multisensory cube as the stop-signal. This change in procedure could have impacted the results in an unintended way as the sudden onset of the LED light and, therefore, general change in luminosity in an otherwise dark environment may have been a more effective stop-signal (akin to a change in loudness for an auditory stop-signal; Ramautar et al. 2006). Thus, it is possible that the change in location may not be the sole contributor to the performance improvement from study 1 to 2 and 2 in the visual stop-signal condition. Future studies may investigate the effect of differently salient LED stop-signals in different locations by varying the distance to the go-signal continuously. Fifth, although somewhat unlikely, it is possible that this difference was due to potential changes in the testing population between experiments. To further support the present results, future researchers need to replicate the present study with a within-subjects experiment that allows them to test the effect of stop-signal location independent of changes in the tested population. Sixth, all experiments required participants to wear earplugs and they were presented with brown noise. These additional precautions, required to mask the sound of the vibrations, may have influenced the result and led to a general slow-down of participants. Seventh, the sample in all studies consisted of mainly self-identified female individuals. Although there is neuroscientific evidence suggesting differences in brain activation in response inhibition tasks, there were no performance differences between genders in those studies (Li et al. 2009, 2006). Eighth, the utilization of the staircase procedure to adjust SSDs is not without risk and can result in a slowing bias and strategic behavior by participants (Tran et al. 2023). This may partially explain the correct Go-RT increase across sessions in study 1 and the order effects of study 2. Ninth, notably, none of these studies was preceded by a psychophysics experiment to equally match all stimuli used. However, the effects obtained in the present study seem robust even with arguably suboptimal stimuli. Future studies may aim to explore the effects of ideally matched or mismatched stimuli.

### Conclusion

In summary, two main conclusions can be drawn from our data. First, a mismatch between stop- and go-signal modality can increase reactive stopping performance, and tactile stop-signals are processed more effectively compared to visual stop-signals in an otherwise visual task. Second, disentangling stop- and go-signal location increases stopping performance even if stop- and go-signals are within the same modality.

## Data Availability

All data are publicly available under https://osf.io/f5bkp/.

## Footnotes

1 There are other tasks such as the go/no-go task that measure inhibitory control. While both tasks involve inhibitory control, the Go/No-Go task focuses on withholding a response when a specific stimulus is presented, whereas the Stop Signal Task examines the ability to inhibit an already initiated response when prompted by a stop-signal. However, while both also rely on similar neural generators, the key difference is the time between go and stop signal (0 for the go/no-go and ~200–400 ms for the SST) (Raud et al. 2020).

2 Order was incorporated into the model as a control factor. No significant effects were expected due to the counterbalancing procedure.
